# Supplementary material for: Identification of an immune gene expression signature associated with favorable clinical features in Treg-enriched patient tumor samples
Source: NPJ Genom Med. 2018 Jun 13;3:14. doi: 10.1038/s41525-018-0054-7 (PMC5998068; doi:10.1038/s41525-018-0054-7)
Supplement: Supplementary file 1 — Supplemental Data [file 41525_2018_54_MOESM1_ESM.pdf]

Supplemental Table 1

	Observed Counts (P = 0.104; x^2= 7.69)		Expected Counts	
Cancer Type	Cluster1 (n = 57)	Cluster2 (n = 78)	Cluster1	Cluster2
BLCA	8	10	7.6	10.4
LUAD	14	23	15.6	21.4
PAAD	9	24	13.9	19.1
SKCM	15	9	10.1	13.9
STAD	11	12	9.7	13.3

Chi-squared = 7.69
P-value = 0.104

Supplemental Table 1. Chi-squared test of independence between tumor anatomical location and counts comprised by each cluster produced by k-means analysis of 32-gene signature.

Supplemental Table 2

	Observed Counts (P = 0.250; x^2 = 5.39)		Expected Counts	
Cancer Type	Resistant patients in Cluster1 (n = 17)	Resistant patients in Cluster2 (n = 45)	Resistant patients in Cluster1	Resistant patients in Cluster2
BLCA	2	7	2.5	6.5
LUAD	5	8	3.6	9.4
PAAD	3	16	5.2	13.8
SKCM	6	7	3.6	9.4
STAD	1	7	2.2	5.8

Chi-squared = 5.39
P-value = 0.250

Supplemental Table 2. Chi-squared test of independence between tumor anatomical location and resistant patient cohort produced by k-means analysis of 32-gene signature (resistant patients from cluster1 vs resistant patients from cluster2).

Supplemental Table 3

Immune Cell Type	Cluster1 treatment resistant patient mean abundances (n = 17)	Cluster2 treatment resistant patient mean abundances (n = 45)	t-statistic	P-value
Macrophages M0	0.0897	0.1905	-4.0085	0.00017
T cells CD8	0.1450	0.0803	2.4102	0.02545
T cells CD4 memory activated	0.0192	0.0065	2.3267	0.02980
B cells naive	0.1258	0.0676	2.0702	0.04963
Mast cells activated	0.0000	0.0057	-2.0182	0.04983
Mast cells resting	0.0285	0.0493	-1.9430	0.05763
Neutrophils	0.0013	0.0101	-1.9269	0.06016
NK cells activated	0.0044	0.0141	-1.8654	0.06740
NK cells resting	0.0262	0.0355	-1.2150	0.23006
Eosinophils	0.0000	0.0004	-1.2053	0.23469
Macrophages M2	0.1640	0.1951	-1.1355	0.26137
Monocytes	0.0264	0.0416	-1.0362	0.30458
Macrophages M1	0.0687	0.0551	1.0105	0.31998
T cells CD4 naive	0.0027	0.0002	0.9263	0.36793
T cells follicular helper	0.0442	0.0355	0.9003	0.37328
T cells CD4 memory resting	0.1697	0.1465	0.7722	0.44610
Plasma cells	0.0405	0.0520	-0.6954	0.49026
Dendritic cells activated	0.0154	0.0213	-0.6840	0.49743
T cells regulatory (Tregs)	0.0161	0.0127	0.5372	0.59626
B cells memory	0.0344	0.0255	0.5159	0.60993
Dendritic cells resting	0.0097	0.0087	0.1406	0.88949

Supplemental Table 3. RNA-seq tumor sample deconvolution to immune cell type abundances (absolute value means) between treatment-resistant patients from cluster1 and cluster2 in discovery data.

Supplemental Table 4

Immune Cell Type	cluster1 mean abundances (n=332)	cluster2 mean abundances (n=294)	t-statistic	P-value
T cells CD8	0.135	0.069	9.915	2.15E-21
B cells naive	0.085	0.036	7.179	2.61E-12
T cells CD4 memory activated	0.021	0.009	6.600	9.11E-11
Macrophages M0	0.125	0.184	-5.976	4.19E-09
Macrophages M1	0.070	0.049	5.735	1.52E-08
Macrophages M2	0.168	0.215	-5.278	1.92E-07
T cells follicular helper	0.048	0.033	5.129	3.89E-07
Dendritic cells activated	0.016	0.029	-4.652	4.18E-06
T cells regulatory (Tregs)	0.021	0.013	4.255	2.45E-05
Neutrophils	0.003	0.011	-3.923	0.0001
Mast cells resting	0.032	0.046	-3.781	0.0002
Plasma cells	0.047	0.068	-3.431	0.0007
B cells memory	0.038	0.021	3.141	0.0018
Mast cells activated	0.004	0.011	-2.794	0.0055
Monocytes	0.022	0.030	-2.717	0.0068
Dendritic cells resting	0.009	0.014	-2.466	0.0140
T cells CD4 naive	0.002	0.001	1.375	0.1697
NK cells resting	0.038	0.035	1.151	0.2500
NK cells activated	0.008	0.010	-1.131	0.2585
Eosinophils	0.001	0.001	-0.919	0.3587
T cells CD4 memory resting	0.149	0.146	0.376	0.7067

Supplemental Table 4. RNA-seq tumor sample deconvolution to immune cell type abundances (absolute value means) between clusters derived from 32-gene signature in TCGA validation set (n = 626).

Supplemental Figure 1

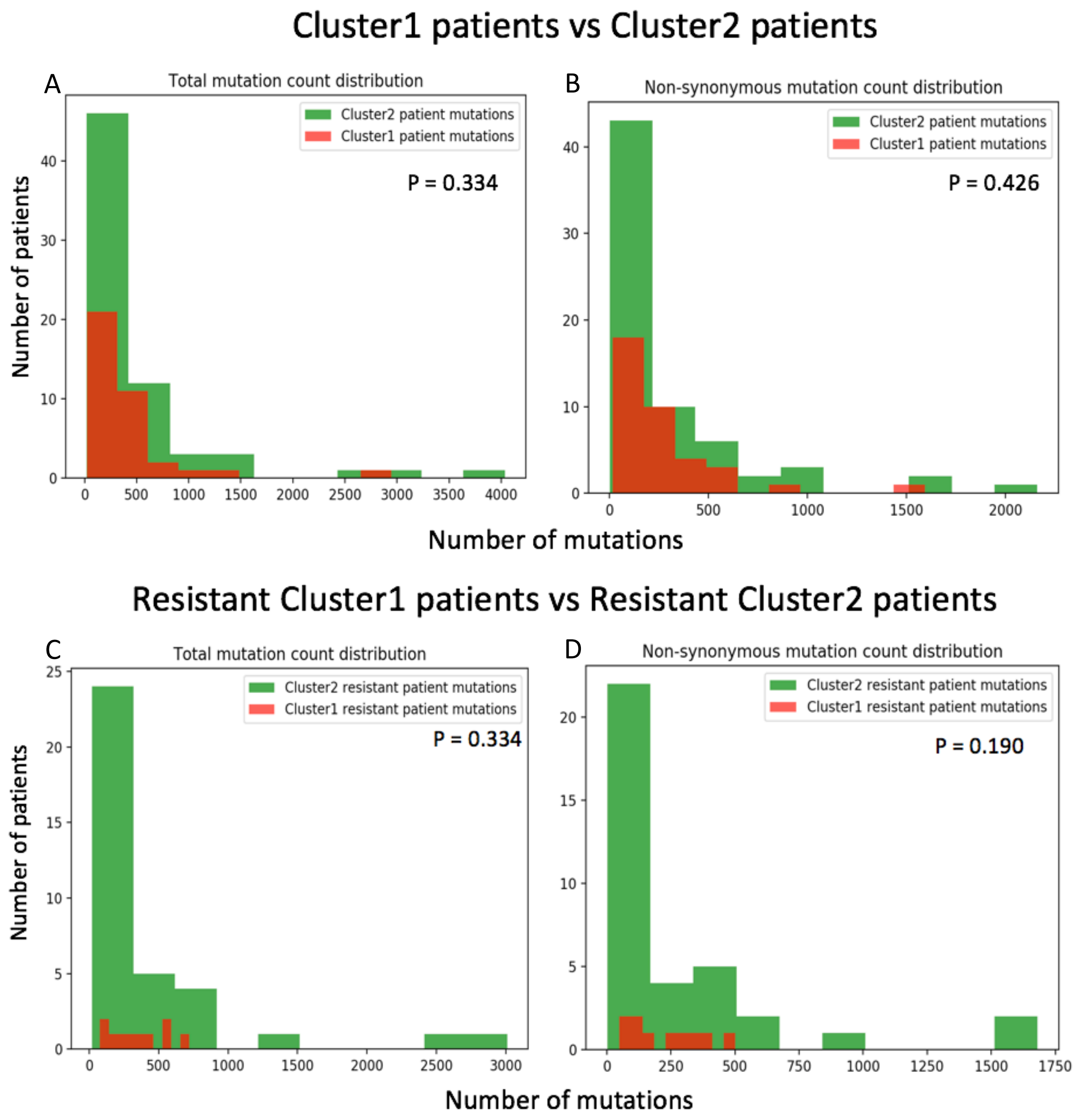

Supplemental Figure 1. Mutation count distributions between cohorts. **(A)** Total mutation counts between cluster1 patients and cluster2 patients. **(B)** Non-synonymous mutation counts between cluster1 patients and cluster2 patients. **(C)** Total mutation counts between cluster1 patients resistant to treatment and cluster2 patients resistant to treatment. **(D)** Non-synonymous mutation counts between cluster1 patients resistant to treatment and cluster2 patients resistant to treatment.

Supplemental Figure 2

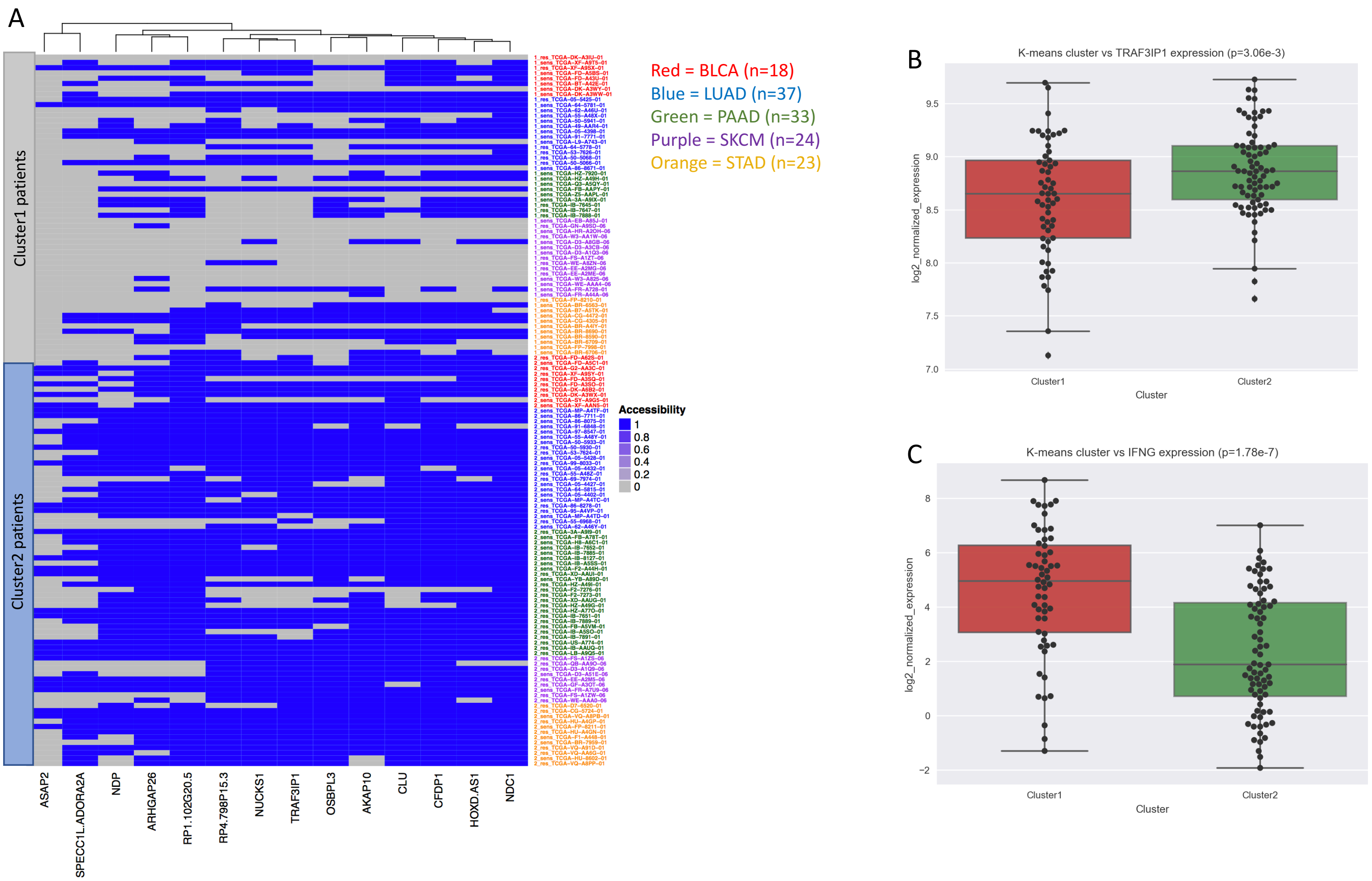

Supplemental Figure 2. Patient tumor sample DNA accessibility analysis. (A) sites within 14 genes were predicted to be uniquely enriched for accessibility in cluster2 patients (acceptable P < 5.8e-7). (B) Differential TRAF3IP1gene expression between cluster1 and cluster2 patients (P < 3.06e-3). (C) Differential IFNG gene expression between cluster1 and cluster2 patients (P < 1.78e-7).

Supplemental Figure 3

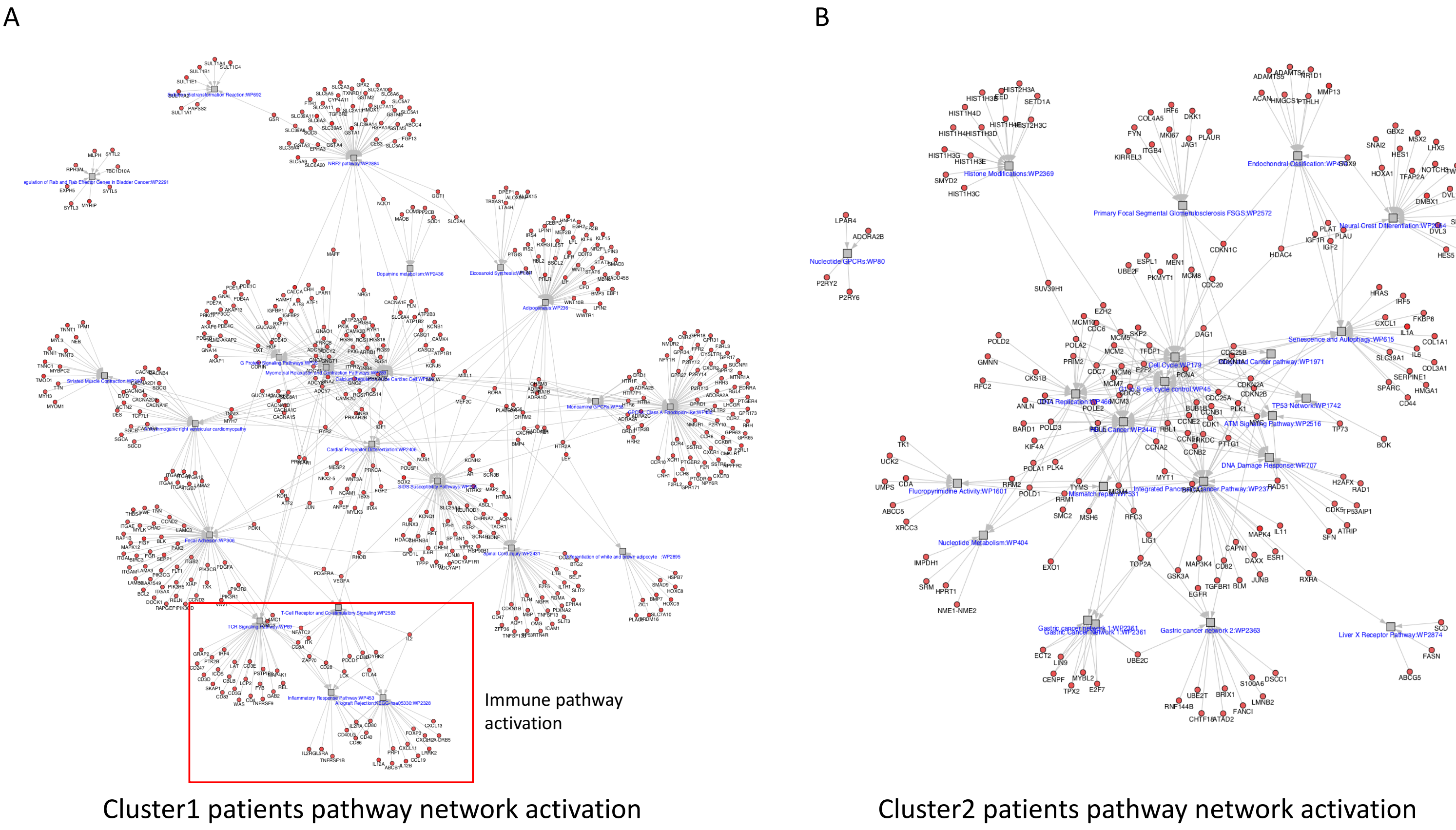

Supplemental Figure 3. Patient cluster pathway network analysis. (A) Upregulated pathways in cluster1 patients vs. cluster 2 patients, with associated upregulated genes (red) and squares (grey) representative of a given upregulated pathway. Immune pathways are boxed in red. (B) Upregulated pathways in cluster2 patients vs. cluster 1 patients, with associated upregulated genes (gene) and squares (grey) representative of a given upregulated pathway.
